# Supplementary material for: Modelling proteins’ hidden conformations to predict antibiotic resistance
Source: Nat Commun. 2016 Oct 6;7:12965. doi: 10.1038/ncomms12965 (PMC5477488; doi:10.1038/ncomms12965)
Supplement: Supplementary Information — Supplementary Figures 1 - 4 [file ncomms12965-s1.pdf]

Supplementary Figures

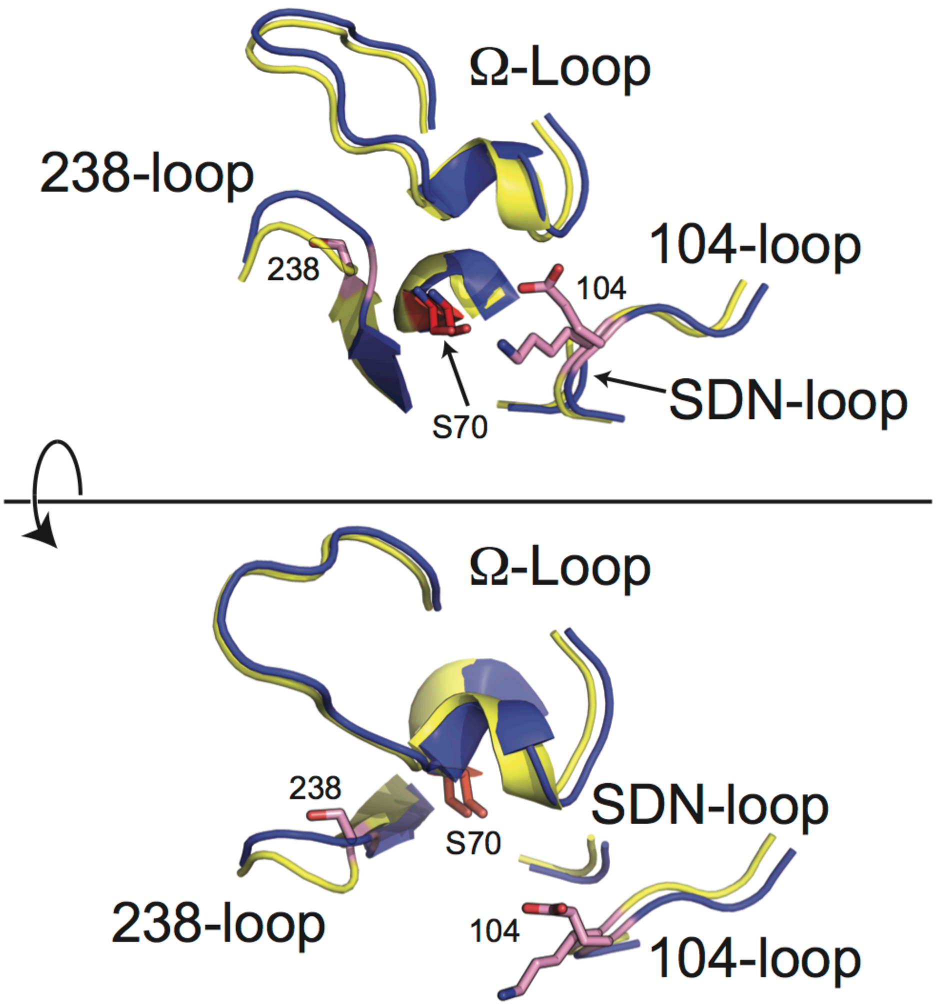

Supplementary Figure 1. Overlay of important loops in the active sites of TEM-1 (blue, PDB 1BTL) and TEM-52 (yellow, PDB 1HTZ) reveals subtle conformational differences, particularly in the 238-loops.

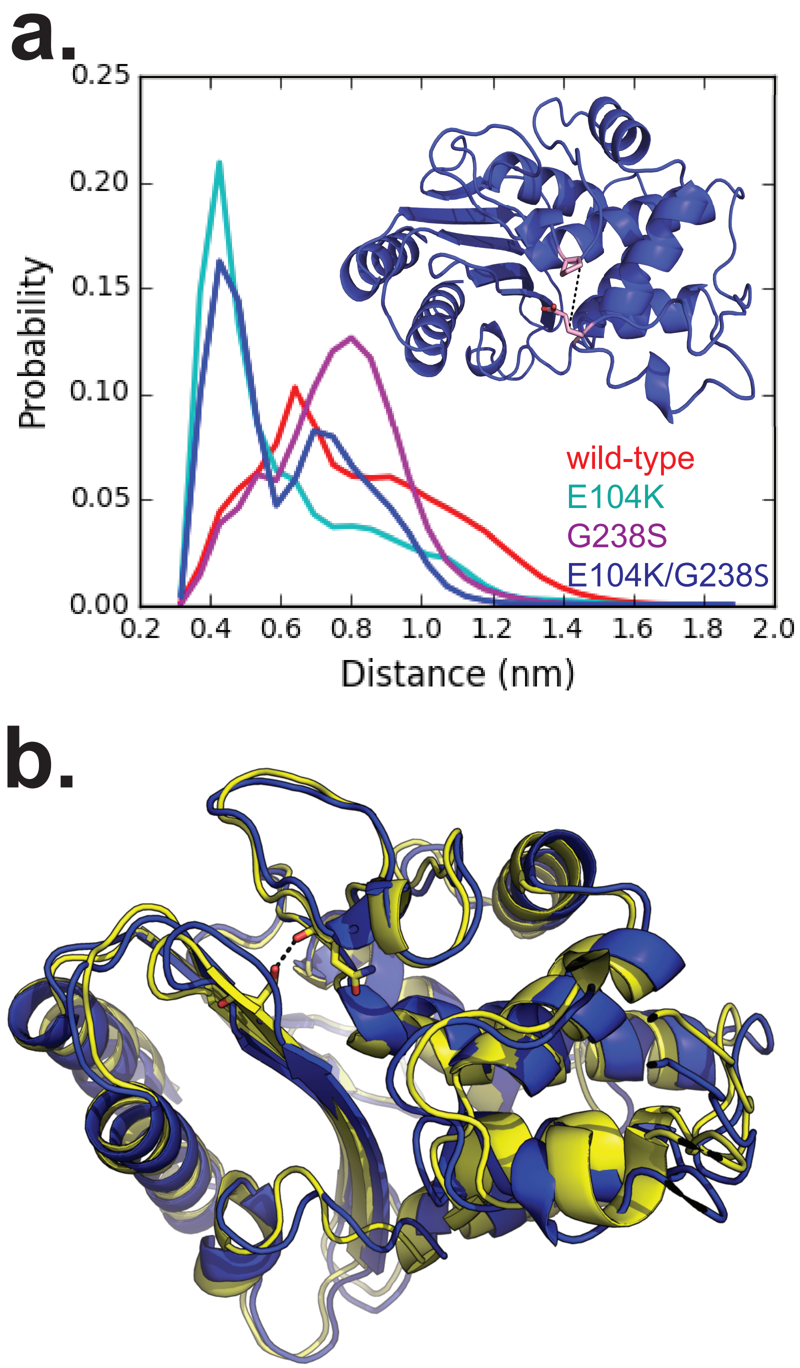

Supplementary Figure 2. E104K and G238S substitutions restrict motion in the Ω-loop. (a) The distribution of distances between position 104 and 167 of the Ω-loop (inset) shows that states with a short distance are more probable in variants containing the E104K substitution. (b) A representative structure from simulations containing the G238S substitution reveals a hydrogen bond between the side chain of S238 and backbone carbonyl of N170.

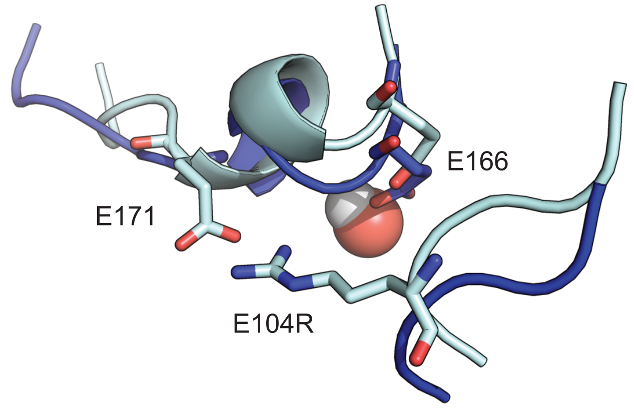

Supplementary Figure 3. One representative structure from the E104R/G238S variant (cyan) shows a salt-bridge between R104 and E171 in the Ω-loop. This causes displacement of the loop relative to wild-type (blue), altering the position of a key residue (E166) that is important for coordinating the catalytic water. We hypothesize this non-productive conformation accounts for E104R/G238S's lower activity (k_{cat}) against all substrates, particularly relative to E104K/G238S and E104M/G238S.

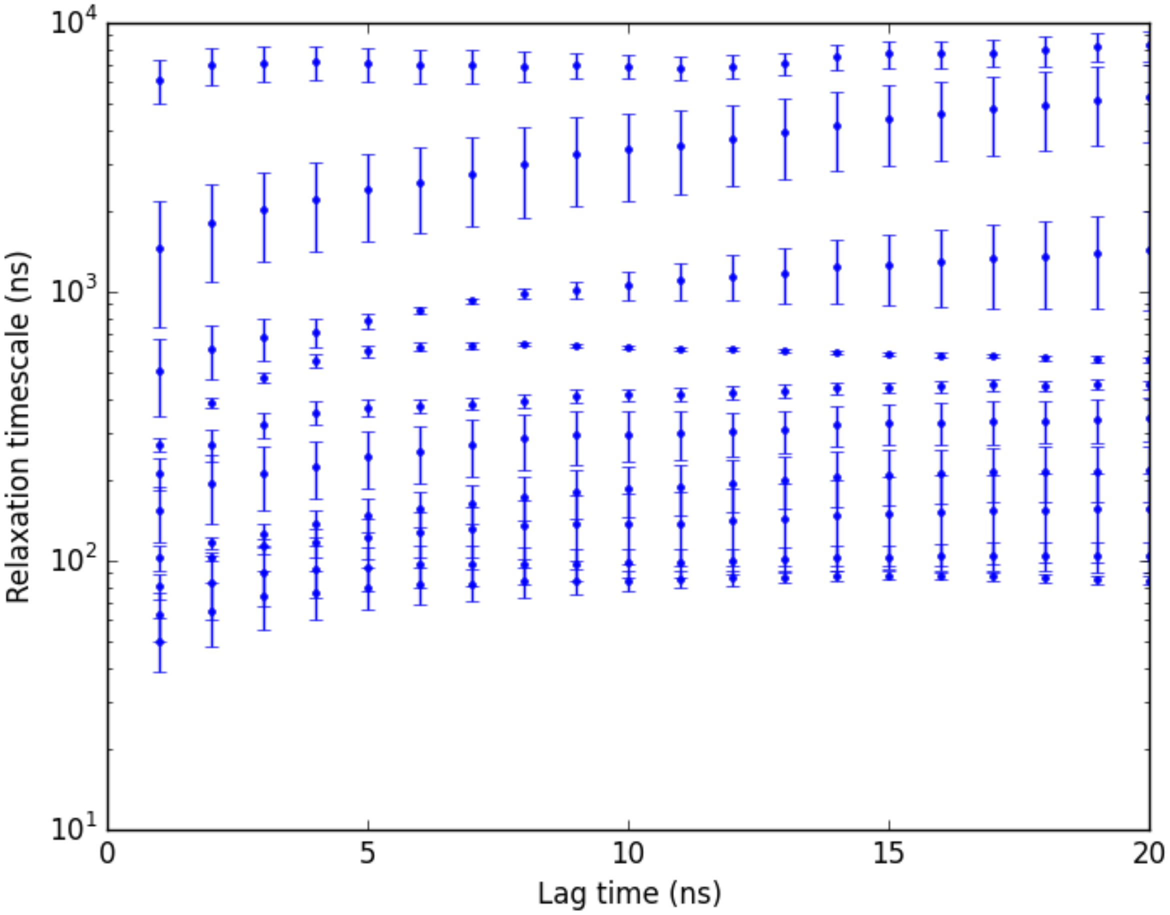

Supplementary Figure 4. Relaxation timescales for TEM-1 establish the model satisfies the Markov assumption. Relaxation timescales are shown for lag times from 1 to 20 ns. Both axes are in ns. The flatness of the plots suggests the model satisfies the Markov assumption for lag times as small as 1 ns. The relaxation timescales for the other variants are qualitatively similar.
